# Chronic nail biting, orthodontic treatment and Enterobacteriaceae in the oral cavity

**DOI:** 10.4317/jced.56059

**Published:** 2019-12-01

**Authors:** Alagesan Chinnasamy, Karthikeyan Ramalingam, Pallu Chopra, Vidhya Gopinath, Gyan-Prakash Bishnoi, Gurveen Chawla

**Affiliations:** 1 Population Oral Health, Melbourne Dental School, The University of Melbourne, Australia; 2 Oral Pathology and Microbiology, Surendera Dental College & Research Institute, Rajasthan, India; 3 Saveetha Dental College, Chennai, India

## Abstract

**Background:**

Chronic nail biting is common in children and young adults. Auto inoculation of environmental pathogens can manifest as infection in distant organs. Multi-drug resistance gram negative bacteria are on the rise globally. Several of the foodborne bacteria fall within the Enterobacteriaceae family but very few studies have explored these microbes in the oral cavity of children with chronic nail-biting habit or orthodontic treatment. The study aims to investigate oral load of Enterobacteriaceae in children with chronic nail-biting habit and or those undergoing orthodontic treatment.

**Material and Methods:**

150 children (no nail-biting n=30, nail biting n=60, fixed orthodontic treatment n =30 and a combination of fixed orthodontic appliance use and nail-biting habit n =30) were assessed for culture based microbiological investigation. The concentrated oral rinse technique was used. The rinse was inoculated in MacConkey’s and Blood Agar. The gram stained culture was subjected to biochemical tests for sub-species identification using Biomerieux Vitek 2 Compact Automated Microbiological Analyzer. Fisher’s exact and Kruskal Wallis with post hoc analysis using Dunn method was performed to test association and difference between groups.

**Results:**

Enterobacteriaceae was positive for 72% of the children. Of them, nail biting or orthodontic treatment group comprised 89%. Those with a combination of nail biting and undergoing orthodontic treatment exhibited highest CFU/ml and those without nail biting or orthodontic treatment exhibited the lowest. Three of the four organisms isolated tested positive in the orthodontic treatment group. *E. coli* was positive in 38% of the children while *Klebsiella* and *E. cloacae* were isolated exclusively in the orthodontic treatment group.

**Conclusions:**

Chronic nail biting or the use of fixed orthodontic appliances is associated with higher incidence of Enterobacteriaceae in the oral cavity. Oral health professionals play an important role in preventing multi drug resistance infectious diseases.

** Key words:**Enterobacteriaceae, nail-biting, Onychophagia, orthodontic treatment.

## Introduction

Onychophagia or nail biting is an oral compulsive behavioural disorder particularly common in children and young adults. Its prevalence is estimated to be between 6 to 45% (1-3). Although habitual nail biting is often considered to be harmless and wanes off as age advances, infrequently they have been associated with a range of psychological (1,4), dental (5-9), systemic infections and diseases (10,11).

The oral cavity harbours a diverse set of more than 1,000 bacterial species (12). Nail biting increases the carriage of several opportunistic environmental microorganisms into the oral cavity. Among those include Enterobacteriaceae, a group of facultative anaerobic gram-negative bacteria that are thought to be transient or non-resident members of the oral cavity in healthy individuals (13). Some potentially pathogenic intestinal microorganisms of the Enterobacteriaceae family like *Escherichia Coli* (*E. coli*) and Enterobacter have been noted in the oral cavity of nail biters (14,15).

Since most of the orthodontic treatment is carried out in school age children removal of food debris becomes challenging in difficult to reach sites in and around the appliances that are in close proximity to the oral tissues. This acts as a reservoir favouring microbial growth. With less than optimal hand hygiene compounded with the habit of nail biting, children can accidentally transport environmental pathogens to the oral cavity. Studies have identified children with the combination of nail biting and the use of orthodontic appliances exhibit significantly higher incidence of Enterobacteriaceae than those with nail biting alone (16,17). Repetitive habitual nail biting compounded with less than optimal oral hygiene in children’s receiving orthodontic treatment can carry the microbial loads to distant organs like the lungs, intestine and present as systemic manifestation altering the balance between health and disease (18-20). The test hypothesis is that chronic nail-biting habit or orthodontic treatment in isolation or in combination does not increase oral Enterobacteriaceae count. As such, this study aims to investigate oral load of Enterobacteriaceae in children with chronic nail-biting habit and or those undergoing orthodontic treatment compared to those with no nail biting or no orthodontic treatment.

## Material and Methods

A culture based microbiological investigation was carried out among school children between six and 17 years attending the outpatient clinic at Surendara Dental College & Research Institute, India. An Institutional Ethical Committee approval (SDC/IEC/2016/013) was obtained for this research work. Participating children and accompanying parents were informed of the study and formal consent was obtained. Those with any known medical illness or antibiotic therapy in the past three months, uncooperative or apprehensive, thumb sucking habit and those on antiseptic mouth wash were excluded. The study comprised of 150 individuals of both gender and sub divided into four independent groups, group 1 is the control group (n=30) comprised of individuals with neither nail biting habit nor orthodontic appliance use, group 2 (n=60) with only nail biting habit, group 3 (n=30) with fixed orthodontic multiple bracket appliance use for a minimum of three months period and group 4 (n=30) with a combination of chronic nail biting habit along with fixed orthodontic multiple bracket appliance use for three months or more. All the groups were mutually exclusive where participants cannot be in more than one group at any given time.

The concentrated oral rinse technique described by Samaranayake *et al.* (21) to detect oral Candidal species was adopted for culturing oral Enterobacteriaceae. In brief, all the subjects were provided a 10 ml phosphate buffered saline in a sterile container and advised to rinse their mouth for a minute. The rinse is then collected in a sterile container and taken for microbiological analysis. The oral rinse was then centrifuged at 17,000 g (12,500 rpm) for ten minutes, the supernatant was discarded, and the deposit was resuspended in 1ml of phosphate buffered saline to yield the concentrated rinse. A full loop of concentrated rinse was inoculated in MacConkey’s (M082 Hi-media Labs, India) and Blood Agar (UNSPC code- 12352200) culture media plates using Standard streak plate method. The streaked plates were incubated for 24 hours and the culture was examined for Enterobacteriaceae growth in pink and white coloured colonies. The calculation of the Colony Forming Unit per millilitre (CFU/ml) is based on the formula CFU/ml = (number of colonies multiplied by the dilution factor) divided by the volume of culture plates.

**Figure 1:**
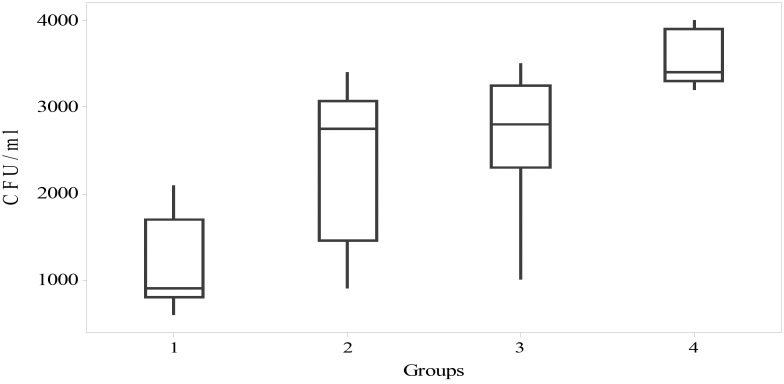
CFU/ml (optional).

A small amount of lactose fermenting colonies was transferred aseptically with inoculating loop and smears were made. The culture was gram stained using conventional protocol for visualization under a microscope for gram-negative bacilli. Biochemical tests were performed as per manufacturer instruction for Enterobacteriaceae and sub-species specific microganisms of *Escherichia coli*, *Enterobacter areogenes*, *Klebsiella* and *Enterobacter cloacae* with the help of Biomerieux Vitek 2 Compact Automated Microbiological Analyzer10 (Fig. 1: VITEK 2 Compact system).

Descriptive statistics with frequencies and percentages were used to summarize sociodemographic and dependent variables. To test the association between nail biting, orthodontic treatment and Enterobacteriaceae a Fisher’s exact test was performed. To identify the difference between groups Kruskal-Wallis test and post hoc analysis using the Dunn method (22) was performed. The *p* value was corrected for multiple testing with Bonferroni adjustment. The Kruskal-Wallis test was preferred since the assumption for normality was violated when Shapiro-Wilk’s test identified *p* < .05 when Colony Forming Unit per millilitre (CFU/ml) for the four groups was assessed. The confidence interval was set at 99% and significance level at 0.01 for all the tests and the analyses was performed using Statistical Package for the Social Sciences software (SPSS for Windows, Version 24, Chicago, IL). A priori sample size was determined using GPower with the alpha set at 0.01 and power at 0.95. A large effect size (f2= 0.40) was used for the analysis. Based on these assumptions the minimum number of participants required for the analysis was determined as 148.

## Results

Summary of the descriptive statistics is presented in  Table 1 and  Table 2. Approximately, 69% of the participants were males. The mean age was 12.7 (SD 3.4) for males and 13 (SD 2.7) for females. No significant gender difference was observed for Enterobacteriaceae test results. Overall, Enterobacteriaceae was positive for 72% (n= 108) of the children. Of them, 89% (n=96) were in the nail biting or orthodontic treatment group. Among those tested positive (n= 108) for Enterobacteriaceae 88.9% (n=96) were in the nail biting and orthodontic treatment group compared to 11.1% (n=12) in the non-nail-biting and non-orthodontic treatment group. More number of children tested positive for Enterobacteriaceae in the orthodontic treatment group (85%) compared to 63% in the non-orthodontic treatment group and this difference was statistically significant when assessed by Fisher’s exact test with a *p* <0 .01. Further, a significant association for Enterobacteriaceae in those with nail biting and or orthodontic treatment group compared to those with no nail-biting habit and or not receiving orthodontic treatment as assessed by Fisher’s exact test, *p* < .01.

**Table 1:**
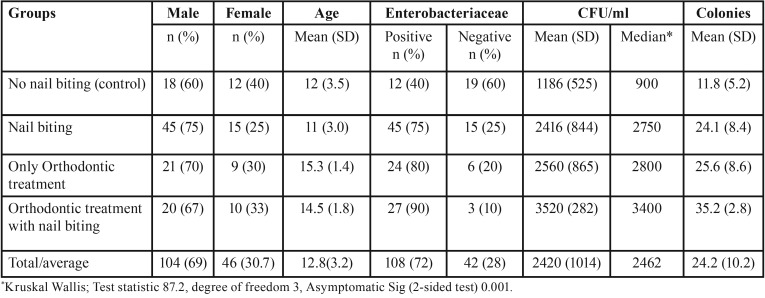
Descriptive statistics of participants and Enterobacteriaceae by groups.

**Table 2:**
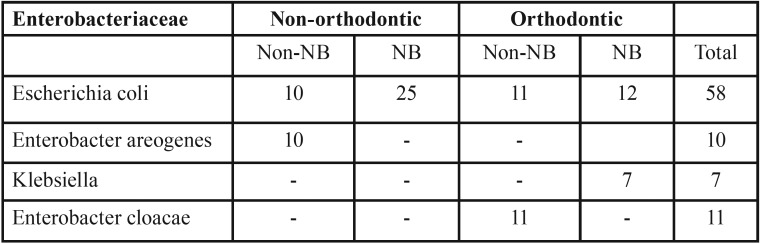
Isolation frequencies of specific opportunistic bacteria.

The Kruskal-Wallis test was used to identify if significant differences existed in the distribution of CFU/ml values in the four groups of participants. Distributions of CFU/ml scores were not similar for all groups, as assessed by visual inspection of the boxplot (not presented in the figure) The results of the post hoc pairwise multiple comparisons are presented in  Table 3. The mean rank of CFU/ml values identified statistically significant difference in five of the six pairwise multiple comparison χ2(3) = 87.2, *p* <.01. The only instance where post hoc analysis identified no statistically significant difference was between nail biting and orthodontic treatment in isolation.

**Table 3:**
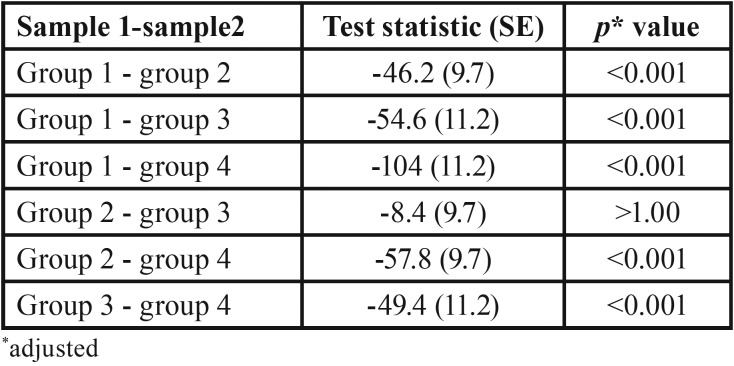
Post-hoc pairwise multiple comparison.

The isolated frequencies of the four Enterobacteriaceae species are presented in table 2. Overall, 39% (58/150) of the subjects tested positive for the four organisms. This implies that 54% (58/108) of the Enterobacteriaceae positive also tested positive for one of the four species of facultative opportunistic gram-negative bacilli. The proportion of those tested positive for the four species of the Enterobacteriaceae was the same for the orthodontic 38% (23/58) and non-orthodontic group 38% (35/58). Three of the four organisms isolated tested positive in the orthodontic treatment group compared to two in the non-orthodontic treatment group. All the 58 subjects tested positive for *E. coli*. Of them, 83% (48/58) were either in the nail-biting group or receiving orthodontic treatment. *Klebsiella* and *E. cloacae* were isolated exclusively in the orthodontic treatment group. Of them Klebsiella was isolated in those with no nail-biting habit but *E. cloacae* were present in those with nail biting habit while receiving orthodontic treatment. *E. aerogenes* was neither present in the nail-biting group nor orthodontic treatment group.

## Discussion

Our study is the first to explore oral load due to nail-biting and the use of orthodontic appliances in healthy children. Our primary interest is to identify the difference in the microbial load and the number of participants in our study is more than any previously reported studies on nail biting and orthodontic treatment. Despite that they are not sufficiently powered to estimate the prevalence due to challenges with sample size and selection. Sedgley and Samaranayake (1994) reported practical difficulties in identifying the prevalence of Enterobacteriaceae due to small sample numbers, and methodology employed (13).

Our findings are consistent with previous research where Enterobacteriaceae in the oral cavity were significantly higher in nail biters compared to non-nail biters (14,15). The results demonstrated higher CFU values for Enterobacteriaceae was associated with the use of fixed orthodontic appliances or the habit of nail biting. The number of people tested positive for Enterobacteriaceae was eight times higher in those with either nail biting habit or orthodontic treatment compared to the control group (non-nail biting). In the four specific microorganisms investigated *E. coli* was predominant and was observed in 39% (n = 58/150) of all the children and 58% (n= 58/108) of all those tested positive for Enterobacteriaceae. Despite the higher proportion of *E. coli* present in nail biters and orthodontic appliance users individual susceptibility was not observed for either of these two groups. However, *Klebsiella* was exclusive to those with a combination of nail biting and orthodontic treatment. Similarly, *E.cloacae* was observed exclusively in those receiving orthodontic treatment with no nail biting habit. Our findings are different from those observed by Kamal and Bernard (23) where *E. coli* and Enterobacter were isolated in children with nail biting habit and *Klebsiella* in the control group.

Nail biting has been associates with several dental and medical conditions. Transfer of oral microbes to the gut through the saliva is not common. A report published in Genome Biology by Segata *et al.* (24) stated The Human Microbiome Project established 45% overlap between oral and stool bacteria. Nail biting increases the risk of accidental inoculation of environmental organism into the oral cavity and the risk is particularly high in children do not wash their hands properly. The relatively immature immune system does not offer adequate protection as in adults and food-borne environmental pathogens can gain entry to the oral cavity when good hand hygiene is unlikely to be optimal. This makes them particularly vulnerable for Enterobacteriaceae infection that is becoming increasingly ineffective to routine antibiotics

Most of the literature on Enterobacteriaceae have focussed on antimicrobial resistance. Commonly used antimicrobials have become ineffective with the emergence of several multi drug resistance Enterobacteriaceae, globally (25-27). The extended spectrum beta lactamase producing Enterobacteriaceae is the most common cause of this public health concern. *E. coli*, *Klebsiella* pneumoniae and Enterobacter species are notoriously known for their pathogenic potential and have been consistently demonstrated the highest prevalence of betalactamase production in children (28,29). The extended-spectrum β-lactamase-associated infections are reported to have four times higher mortality and enduring bacteraemia compared to those without this infection (30). A systematic review and meta-analysis of multi drug resistance *E. coli* in asymptomatic children identified a pooled prevalence of 28.6% to 37.7% for Organisation for Economic Cooperation and Development (OECD) countries and 67.2% to 81.3% for non-OECD countries(27). South-East Asia and Western Pacific regions hold 4.3 of the 7.7 billion world population and that includes the regional heavy weight China and India where antimicrobial resistance for extended-spectrum β-lactamase-producing bacteria in children is particularly high (28).

## Conclusions

Nail biting or the use of fixed orthodontic appliances is associated with higher incidence of Enterobacteriaceae in the oral cavity that can influence the quantity and quality of oral microbiota that have important implication in systemic diseases and conditions. Oral health professionals must ensure that habitual nail biting is recorded in the patient medical and dental history and every effort should be made to ensure the habit is discouraged. Children and accompanying parents should be informed of the adverse consequence in every follow-up visit. The importance of maintaining good hand and oral hygiene should be emphasized at every available opportunity during orthodontic treatment.
